# Moving academic conferences online: Understanding patterns of delegate engagement

**DOI:** 10.1002/ece3.7251

**Published:** 2021-02-15

**Authors:** Cassandra L. Raby, Joah R. Madden

**Affiliations:** ^1^ Association for the Study of Animal Behaviour Leeds UK; ^2^ Washington Singer Labs Centre for Research in Animal Behaviour College of Life and Environmental Sciences University of Exeter Exeter UK

**Keywords:** face‐to‐face, networking, scientific meetings, social media, virtual

## Abstract

Scientific conferences are a key component of academic communication and development. During the COVID‐19 pandemic, in‐person conferences are rapidly moving online, yet these virtual events may not provide the same opportunities as in‐person conferences. If virtual meetings are to continue to provide effective communication and networking between researchers and stakeholders, they must be adapted to increase delegate engagement and enthusiasm. Here, we present a case study of a recent medium‐sized online conference. We assessed the behavior and engagement of delegates with different components of the meeting using data from website analytics and postconference surveys. Behavior was variable across the available events; talks were particularly popular but engagement with social and networking opportunities was variable. Our conclusions have been summarized in six recommendations to support future online conference organizers in enhancing engagement with their events.

## Graphical abstract

Here, we present a case study of a recent medium‐sized online conference. We assessed the behavior and engagement of delegates with different components of the meeting using data from website analytics and postconference surveys. Behavior was variable across the available events, with talks being particularly popular but engagement with social and networking opportunities was variable.

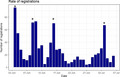

## 1 INTRODUCTION

Academic and scientific conferences are necessary for the dissemination of research, for networking, and to support professional development (Rowe, 2018; Yoo & Chon, 2008). Communication between researchers and other stakeholders occurs formally at talks and poster sessions, and informally at social events, while professional development usually occurs through workshops and mentoring events. Traditionally, conferences have been held as in‐person meetings, and with the increase in international collaborations, delegates travel across the globe to attend these events. Yet, the ethics of conferences have been called into question (e.g., Fraser et al., 2017; Holden et al., 2017). If these events are so integral to scientific development should we accept that they exclude participation of researchers with limited funding? Should we accept that conference travel results in such a large carbon footprint? Alternatively, online conferencing has provided an option for researchers to connect without the same commitment to cost, time, and carbon impact (e.g., Raby & Madden, in review). Online conferences started off as a simple email‐based event (Anderson, 1996), and due to the COVID‐19 pandemic has rapidly become more common and integral to scientific communication (Milić et al., 2020; Viglione, 2020). In order to maintain the enthusiasm of online conferencing, it is vital that we take this opportunity to feedback from the events that have been organized during the COVID‐19 pandemic. The key indicator of delegate enthusiasm for online conference material is their engagement during the event, either indicated quantitatively by their accessing different parts of the meeting (e.g., visiting webpages, viewing videos, and joining live forums) and/or more qualitatively by their responses to postmeeting surveys investigating their self‐reported patterns or experiences of engagement with different parts.

This paper provides a case study of one such event which was the first, to our knowledge, in its field (specifically animal behavior). On the July 16, 2020, the Association for the Study of Animal Behaviour (ASAB) held a one‐day online conference, both as a response to the cancellation of their Easter and Summer meetings due to COVID‐19, and as a foray into a more carbon‐friendly alternative to academic meetings. This online conference format is likely to be an increasingly normal situation as scientific interest groups and learned societies consider delivering virtual noncommercial scientific meetings to replace established small‐ to medium‐sized real‐world meetings. Recently, papers and preprints outlining advice and guidance for developing an online conference have become available to support future event organizers (e.g., Busse & Kleiber, 2020; Gichora et al., 2010; Harabor & Vallati, 2020; Lortie, 2020; Roos et al., 2020; Saliba, 2020; Seery & Flaherty, 2020), yet few have explored delegate engagement with these online formats or considered if online conferences should differ from their traditional in‐person counterparts. In order to help future meeting planners with decision‐making, we have reported the patterns of attendance and engagement with our meeting, highlighted where loads may be uneven, and used these data to indicate which components were particularly (un)popular. Additionally, we have supported our findings with postconference feedback from delegates on their motivations to engage with the meeting and self‐reported data on the perceived successes and failures of the meeting. Ultimately, exploration of what does and does not replicate across online and in‐person conferences is necessary if future conference organizers are to produce effecting and engaging meetings online.

### 1.1 Establishing the online meeting format

Since this was the first foray into virtual international meetings by ASAB, and few other scientific organizations had developed online conferences at the time of planning, we did not have a template to follow and so were unsure what content was desirable or deliverable. Consequently, we decided to emulate all the components of an in‐person meeting with virtual equivalents of plenaries (longer invited talks by established researchers); research talks (shorter talks submitted by delegates); an opportunity to question presenters; poster presentations (with the opportunity for delegates to chat with the presenter); professional development opportunities (mentoring of students/early career researchers by established academics or a discussion with a Journal Editor about publication process and strategies); a link to the Society via a President's address; opportunities for advertisers to exhibit wares; professional socializing (the chance to interact with others with similar research interests); and informal socializing (the chance to interact with community members over subjects other than research via communal drinks, quizzes or a dance ceilidh—we did not attempt to emulate the latter).

The online conference had 13 prerecorded talks (3 plenaries and 10 research talks) and a live Q&A (question and answer) webinar at the end of each talk session. Our poster session had 30 posters with Zoom meetings to each of the poster presenters. We offered two professional development and two social activities during the day. The development sessions comprised an opportunity to “Meet the Editor” of the society journal Animal Behaviour where prospective authors could find out more about the submission and review process, and a mentoring session where early career researchers can meet with mentors for guidance and advice. The social events comprised of Science Cafés, where delegates were grouped into meetings based on research interest, and an animal behavior themed quiz. Delegates had to sign up to participate in the social activities prior to the meeting day. See Raby & Madden (in review) for a detailed summary of the conference content and software used. Our approach was to attempt everything (or at least as much as was feasible) and then to prune out elements from future conferences that were poorly received or which did not perform well following feedback from delegates who had used them. Therefore, we collected data on the engagement by the conference delegates to establish the effectiveness of online conferences.

## 2 METHODS

We collected data about the engagement and experience of delegates in two ways. During the meeting, we monitored engagement with the meeting website and the social media account via Wix, Twitter, and Google Analytics. At the end of the meeting, we asked delegates that attended to complete a (anonymous) postmeeting survey (*n* = 66, 14% of attendees) comprising a series of forced choices, free choices, Likert scale agreement scores (7 point scale), and free‐text responses (see Appendix A). We did not offer any inducements to complete the surveys. All data were anonymized automatically, and all conference delegates accepted our privacy policy and terms of conditions for the use of cookies on the website and the use of data when registering.

## 3 RESULTS

### 3.1 Registration and attendance

**FIGURE 1:**
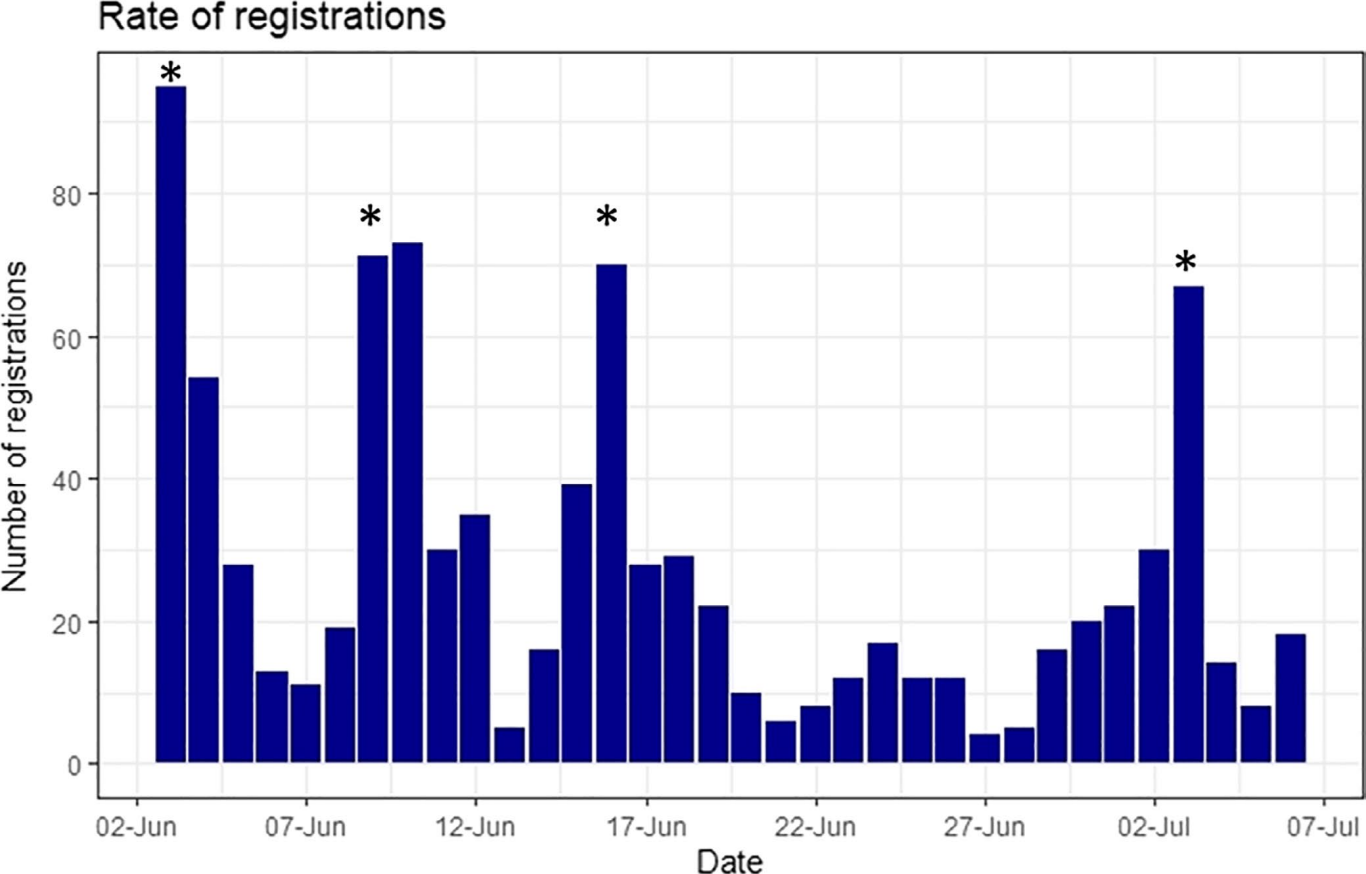
Number of registrations for the online conference every day from when the registration opened (3rd June), until the registration closed (3rd July, extended until 6th July). The * indicate dates where promotional emails and tweets were circulated to ASAB members, and other interested groups (3rd June; 9th June; 16th June; and 3rd July)

Of the 950 people who registered for the meeting, just over half (*n* = 480, 51%) actually “attended” the conference live (i.e., logged on to some part of the website during the conference day). Registration was generally steady across the month that the registration was open for, except for the first day the registration opened where 104 people (11% of delegates) signed up, the highest uptake of any day. Additionally, there were peaks of registration sign ups on days that promotional emails were circulated and on the final day of registration (~70 people each day) (Figure 1). We closed registration on 3rd July to ensure that we had capacity in our platforms to serve all the anticipated delegates. On the day of the conference, most of the traffic was from people that had not registered in advance (first‐time visitors 63.5% vs. returning visitor 36.5%), with the total of 1,380 people accessing the conference website on the conference day.

Because the online meeting was free, it is difficult to make comparisons with many in‐person meetings which generally charge for attendance. However, the ASAB winter meeting, held annually in London, is also free and aimed at the same general audience with a specific animal behavior theme and thus the most viable direct comparison. In 2018, 204 delegates attended that meeting and in 2019, 172 delegates attended.

### 3.2 Engagement with scientific content

#### 3.2.1 Talks and plenaries

For delegates that attended the conference their engagement with the delivered scientific content of the meeting was high. Between 323 and 449 (67%–93%) delegates accessed the talk pages (session 1:449; session 2:348; session 3:323), and 394 (82%) delegates attended the single poster session. This engagement is mirrored in the postsurvey data. Of the postsurvey respondents, 98% watched at least one plenary talk (8% 1 talk; 33% 2 talks; and 58% all 3 talks); 98% watched at least one submitted talk (22% 1–4 talks; 23% 5–8 talks; and 53% 9–10 talks). The talks were also seen as useful and enjoyable by delegates with 61% of postsurvey respondents reporting that plenaries were most useful and 73% reporting that the submitted talks were most useful, while 41% reported that the plenaries were most enjoyable and 59% reporting that the submitted talks were most enjoyable.

Delegates expressed some appreciation of the value that virtual meetings offer for exposure to a wider range of researchers, especially international speakers, with 53% agreeing (scoring 1–3, 12.5% scored 1 “Strongly agree”) that a virtual meeting was more attractive than a real life one for these reasons. We had used this opportunity to invite two plenaries from outside the UK/EU (one United States and one Japan) who we would not normally have been able to provide travel expenses for. Only 23% (scored 1–3, 3% scored 1, “strongly agree”) of postmeeting survey respondents agreed that virtual meetings were less attractive than in‐person meetings because it was harder to convey work via posters or talks. Therefore, the ability of virtual events to invite speakers from international locations without budget limitations serves as an important advantage of online conferences compared to in‐person conferences.

#### 3.2.2 Live Q&A sessions

Compared with watching talks, the interaction by delegates with the scientific presenters was markedly lower. At the post‐talk Q&A sessions, we recorded a maximum of 120 delegates logged in for Session 1 (1 plenary + 5 submitted talks); 75 for Session 2 (1 plenary); and 67 for Session 3 (1 plenary + 5 submitted talks). Our approach, to publish a set of talks and then host all the presenters as a panel for a prolonged chaired Q&A session featuring submitted questions, met with a mixed response. One delegate found that series of talks, lasting for a maximum of 80 min unpunctured by questions, was too much, commenting “no live interaction until the Q&A was a bit depressing ‐ conferences are about live interactions not catching up on Netflix”. However, another appreciated the consequence that multiple presenters could be quizzed simultaneously, commenting “Loved the panel discussions that evolved out of the Q&A ‐ nice to see those active in the field discussing broader questions as well as those relating to each other's studies” and another delegate appreciated the gap between a talk and the opportunity to question the presenter, commenting “it was great to be able to pause and digest the talks, and to have a bit of time to think about them before the Q and A sessions.” Any questions posed to presenters that were not answered during the live Q&A session were posted on the meeting Forum and the presenter was encouraged to respond to them there. This was appreciated by one delegate who commented that “The Forum and chat allows for more questions and answers, and I can imagine more audience interaction. I may actually prefer this over the real thing.”

For postmeeting respondents, delegates disagreed that the virtual meeting was more attractive than an in‐person meeting because it was easier to interact with other researchers and ask questions (60% scored 5–7, 31% scored 7 “Strongly disagree”), or because it was more comfortable to interact with other researchers and ask questions (54% scored 5–7, 26% scored 7 “Strongly disagree”). We checked the validity of these responses by asking respondents to agree with the statement that the virtual meeting was less attractive than a real life meeting because it is harder to interact with other researchers and ask questions (64% scored 1–3, 22% scored 1 “Strongly agree”). However, for at least some delegates, anonymity afforded by virtual questioning was appreciated, evidenced by comments including: “I think for shy people that might want to remain anonymous, having that button on the website is really useful” and “The way the questions was done was also good, as you felt more confident to ask a question anonymously compared to a 'real life' meeting where it is very intimidating”.

#### 3.2.3 Poster sessions

Engagement with viewing posters was high as 95% of delegates viewed at least one poster in detail (72% 1–10 posters; 19% 11–20 posters; and 4% > 20 posters). However, 70% did not chat with any poster presenter (25% chatted with 1–3; 5% chatted with 4 + presenters). In fact, the poster session was deemed least useful (31%) and least enjoyable (38%). For some delegates, this low evaluation of the posters was likely due to technical problems including difficulties zooming in to text‐heavy posters to read them on screen (29% of postmeeting survey respondents reported problems looking at posters) and/or because some links to poster presenters in zoom chat rooms were password protected (36% of postmeeting survey respondents reported problems talking to poster presenters). Because presenters each hosted their own Zoom chatroom that delegates could visit, those who were reliant on the free version of Zoom were restricted to 40 min, whereas we had scheduled the poster session to last one hour meaning that some chatrooms were closed before the session ended. To avoid this, the poster sessions could have been restricted to 40 min. At least some of those delegates that did engage with poster presenters found it rewarding, commenting: “Chatting with Poster presenters in their individual zoom rooms was brilliant ‐ the closest thing to chatting at a real conference.” “It is easier to read an 'online poster' before chatting to the person. In a normal conference it is hard to read with distractions going on.”

### 3.3 Engagement with professional development and social activities

For the “Meet the Editor” event, we had 36 delegates register and 7 were active on the day (19%). For the mentoring event, we had 60 delegates sign up (and six mentors). We arranged 9 Science Café groups based on clustered interests and had 70 delegates sign up for these. Because these last two events operated autonomously (we left members to arrange meeting times and platforms), we have no data on engagement levels. For the quiz event at the end of the day, we had 65 delegates sign up and 30 participate (46%). The engagement with the social dimension of the meeting provoked several free‐text comments indicating a mix of feelings. For some people, online socializing was easier than in‐person: “In a live conference, it is often ‘weird’ to go up to random people and network with them ‐ being put together in a zoom meeting room is better at breaking the ice and initiating conversation”. “It was actually easier to meet new people (I joined the science cafe) then perhaps in an in‐person conference.” However, for some the situation is uncomfortable and they chose not to engage in these aspects of the meeting: “I don't find virtual socialising comfortable, or a meaningful replacement for in‐person activities, and so did not attend these events.”

### 3.4 Engagement with social media

The meeting had an associated Twitter account @ASABvirtual2020, and this had >700 followers. During the day of the meeting, it was continuously staffed to report on the ongoing events and to channel and reflect delegate comments to followers. During the day, it posted 120 tweets, gaining 80,687 organic impressions, 610 link clicks, 163 retweets, 492 likes, and 85 replies. 62% of postmeeting survey respondents used Twitter to follow content and 41% used Twitter to disseminate content. One respondent reported that “I used Twitter a lot through the day and I think that it massively enhanced my personal experience of the day, and this could be used for to replace communications between non‐presenters going forward.”

## 4 DISCUSSION

Here, we have summarized the behavior and engagement of delegates at an online academic conference. We assessed engagement through delegate registrations and retention, through website analytics and monitoring attendance during webinars, and effectiveness of the online conference format, through a postconference survey. Our findings offer suggestions for enhancing future online conferences, such as improving: conference attendance; delegate engagement with scientific and social content; and social media engagement. Online conferences may never fully capture the same experience as in‐person conferences, and it might take time for academics to adjust to a new way of working. For these reasons, we should not assume that a move to virtual conference post‐COVID will become the norm, even though virtual conferences are key to decarbonising academic meetings (Klöwer et al., 2020; Raby & Madden, in review). Therefore, tailoring the format of conferences to suit the preference of delegates should make online conferences more accepted and effective. We suggest that organizers of online events continue to collect feedback from delegates in order to ensure effective and engaging communication within (and outside of) the academic community (also see Arnal et al., 2020; Bilas et al., 2020; Lortie, 2020). One aspect where engagement was high was the quality of scientific exchange (i.e., plenaries and talks); however, the quality of social events and networking may have been compromised (also see Milić et al., 2020).

It is striking that only half of the people who registered for the online meeting actually engaged with the meeting on the day. Without surveying those who registered but did not attend, it is not possible to determine whether this is driven by the lack of financial commitment (and if this pattern holds when the delegates pay at registration), or whether it is motivated by other attitudes to virtual meetings. The disparity between anticipated and realized engagement poses a problem for online meeting organizers who must prepare for and indeed pay for sufficient capacity and bandwidth to facilitate delegates. This may not be a problem for fully funded meetings that charge registration fees, but there is a (growing) expectation that online events should be free (Anderson, 1996; Raby & Madden, in review), so understanding why those registering do not participate is likely to be important. It was equally striking that very many people did not register, yet visited the website on the day of the meeting, presumably to try to engage. There were almost twice as many first‐time visitors as registered delegates on the day. To mitigate this, online conferences could include a limited number of on‐day registrations to meet their predetermined video conferencing and bandwidth capacities. We also found that social media (Twitter) and emails were an effective way to promote the event, with registration increasing on these days. The increased Twitter activity on the day of the event could go some way to explain the increased website activity from first‐time visitors. Indeed, the organizers received several emails on the day of the meeting from people asking to register on the spot. For real life meetings, registration on the day at meetings is unusual and for those meetings that are economically self‐supporting, financially untenable. Again, it would be important to understand whether this expectation that online meetings should be accessible at the last minute is only relevant to free events or applies more generally to meetings that charge.

Unsurprisingly, the highest levels of engagement were seen with the talks and poster sessions. These traditional components of scientific meetings were engaged with by over two‐thirds of delegates. This attraction may arise from the draw of international speakers; however, it is notable that engagement with these elements declined over the day. This could be explained by delegates from eastern time zones ending their work day and withdrawing. However, we think this unlikely because the majority of non‐EU delegates were from North and South America whose time zones might be expected to make them more likely to engage as the day progressed. The decline in engagement over time may have arisen by chance because the titles of talks later in the day were deemed less interesting, or due to demands on the delegates time later in the day, for example, attending a conference from home exposes the delegate to their personal or family obligations. Alternatively, as some delegates reported in survey responses, they may have become fatigued from continuous online viewing. Termed “video call fatigue” or “Zoom fatigue,” online meetings may be more tiring than in‐person talks as it requires more concentration to engage with nonverbal cues, work against technical issues, and be more attentive to online speakers (Fosslein & Duffy, 2020; Saliba, 2020; Wiederhold, 2020) Data collection from additional meetings and a better understanding of viewer behavior is needed.

Although engagement with posters was high, they were deemed to be of little use and not particularly enjoyable. This is surprising, given that poster sessions are near universal features of scientific meetings and so presumed to be of value to delegates by organizers. However, research into in‐person conferences indicate that posters are considered a less important form of presentation by delegates (Rowe, 2018), and that large numbers of poster presentations can be overwhelming and reduce the effectiveness of networking and communication (Rowe & Ilic, 2015). Despite these findings for in‐person conferences, our virtual conference had 60 posters for >400 delegates and provided the best opportunity for networking through an hour of video conferences scheduled for each presenter. Therefore, either preconceptions of poster presentations, or entirely new factors, are causing delegates to consider poster sessions to be less enjoyable. It may be that viewing posters online suffers from the physical decrease in their size, making reading them difficult. This could be rectified by presenters developing poster design elements suitable for screen viewing including reduced text quantity, enlarged fonts, or the incorporation of interactive elements, for example, links that take the viewer to a video, simulation, sound scape, or supporting references. Alternatively, poster sessions in real life are often highly social occasions, often accompanied by refreshments including alcohol. Viewing posters and approaching presenters may be facilitated by this social lubrication, and this may not be possible to emulate virtually. Thought might be given to replicating this social environment in an online poster session.

Perhaps more surprisingly, many delegates appeared to treat the talks and posters as informative rather than discursive and did not engage with opportunities to discuss the work with the presenters. Relatively few delegates (just 14%–25%) attended the Q&A sessions that mimicked the usual post‐talk question sessions. Likewise, only 30% engaged with poster presenters. Either delegates at real life meetings would rather not sit through the habitual questions that follow a talk, or that the flexibility of the online format meant that most delegates are less likely to engage with live content. The low level of engagement was also seen on the Forum where questions that were not answered during the sessions could be posted and the presenters (or others) address them. Such questions and responses attracted just 15–58 views. We had anticipated that an online format would introduce sufficient anonymity to enable questioners who may not have been confident to address presenters in person (e.g., overcome internal barriers for posing questions, Carter et al., 2018). Instead, survey respondents generally reported that the online format made it harder or less attractive to interact with or question presenters.

Engagement with the professional development activities and the social activities that we offered was low. Networking opportunities are cited as being a key driver for the attendance of delegates to in‐person conferences (Rowe, 2018). Despite this, less than 15% of registered delegates signed up for the Science Café or Quiz and perhaps only half of these actually attended. This was surprising because during premeeting discussions with Society members and in postmeeting surveys of delegates, we consistently encountered the view that the loss of social interactions was anticipated to be, or perceived to be, a major downside of online meetings when compared with real life ones. This lack of social engagement was summed up by one respondent: “What is quite missing is the interaction between the many non‐presenting delegates. To my mind, probably about 90% of all communication that occurs at a conference, including initiation of collaborations, casual discussions and generation of research ideas occurs outside of the presenter‐viewer interaction. That is hard to replace.” In real life meetings within the field of animal behavior, many social events are bottom‐up, being spontaneous and focus on small groups of self‐organizing delegates going to a café or pub to continue discussions. Other social events are top‐down, organized events such as football tournaments, dances, banquets, or visits to local sites of natural interest. Our efforts to promote social mixing were top‐down involving “matchmaking” delegates by research interests for café chats or arranging a quiz that delegates could form teams and compete. However, we did provide a web page (the forum) for delegates to initiate social meetings. This was used by only two delegates but commented on 9–16 times, and resulting in a video meeting over lunch. However, this obviously needed to be promoted and expanded, as postconference responses suggested: “Perhaps a 'networking' part of the discussion page, where people could join together who work on similar things and introduce themselves?”; “One suggestion would be to facilitate contacting people during the day [by having] breakout rooms during the lunch or networking events (for people not participating)…. people want to chat to a small group of people (outside of the scheduled events).” So, despite the opportunities, we were still unable to replicate the usual spontaneous engagement which is probably more realistic of in‐person meetings.

Therefore, we recommend that organizers of future meetings explore how to balance the need to plan and provide organized top‐down virtual social meeting opportunities with the provision of support for bottom‐up delegate driven and spontaneous events. One (partial) solution might be to ask delegates prior to the meeting if they wish to organize social events that others may attend and which the organizers could then facilitate and advertise. Alternatively, or additionally, online meeting organizers may want to consider arranging more “spectacular” social events that delegates could attend in order to build a feeling of community engagement. Some alternatives have attempted to use virtual worlds to imitate conference halls and allow people to meet as virtual avatars, which can be done in games such as Second Life or World of Warcraft (Welch et al., 2010). Another route for international conferences would be to hold presentations virtually, and social events can be done more locally within groups (e.g., Abbott, 2020; Case t et al., 2018; Fraser et al., 2017). Either way, we might need to make online conferences more immersive and flexible if they are to continue post‐COVID.

## 5 CONCLUSIONS AND RECOMMENDATIONS

1. The use of emails and social media are essential to promoting conference registration and website engagement.
2. Talks and plenaries are the most attractive sessions of an online conference, and so future conferences should aim to retain this format where possible.
3. For greatest participation keep registration open throughout the conference, however, this needs to be balanced against the ability to budget for the conference or accurately assess the required capacity of websites and video conferencing software.
4. For poster sessions, posters should be in the landscape format and available to download for ease of reading. In addition, there is considerable potential for online posters to integrate wider media (e.g., videos, QR codes) to enhance presentation. Alternatively, consider changing poster sessions to lightning talks.
5. Allow delegates to sign up for the conference organized social and networking events on the day, although this would be difficult to organize there would be greater participation than expecting delegates to sign up in advance.
6. Replicate bottom‐up socializing and events by providing a networking page where delegates can easily contact each other to organize their own meetings.

## CONFLICT OF INTEREST

All authors state that there is no conflict of interest.

## AUTHOR CONTRIBUTIONS

**Cassandra L. Raby:** Data curation (equal); investigation (equal); methodology (equal); writing – original draft (equal); writing – review and editing (equal). **Joah R. Madden:** Data curation (equal); investigation (equal); methodology (equal); writing – original draft (equal); writing – review and editing (equal).

## Data Availability

All data have been archived and made available at https://doi.org/10.5061/dryad.280gb5mp8.

## APPENDIX A

### Postconference questions and options

1. What is your career stage?
  - Early career researcher
  - Other academic role
  - Non‐academic role
  - Senior academic role
  - Student
2. Average number of ASAB meetings that you attend each year?
  - 0
  - 1
  - 2
  - 3
  - N/A
3. Average number of other conferences or workshops that you attend each year
  - Less than 1
  - 1 per year
  - 2–4
  - 5+
  - N/A
4. Where are you based?
  - Africa
  - Asia
  - North America
  - Other EU
  - South America
  - UK
  - Australasia
5. Did you present today?
  - Talk
  - Poster
  - No
6. How many plenaries did you watch?
  - 0
  - 1
  - 2
  - 3
7. How many invited talks did you watch?
  - 0
  - 1–2
  - 3–4
  - 5–6
  - 7–8
8. How many posters did you look at in detail?
  - 0
  - 1–3
  - 4–6
  - 7–9
  - 10+
9. How many poster presenters did you chat with?
  - 0
  - 1–3
  - 4–6
  - 7–9
  - 10+
10. The meeting element that I found MOST useful (tick any)
  - Plenaries
  - Invited talks
  - Posters
  - Mentoring/Science cafes/Meet the Editor
  - Pub quiz/Other socializing
11. Can you briefly explain why?

[free text]

1. The meeting element that I found LEAST useful (tick any)
  - Plenaries
  - Invited talks
  - Posters
  - Mentoring/Science cafes/Meet the Editor
  - Pub quiz/Other socializing
2. Can you briefly explain why?

[free text]

1. The meeting element that I found MOST enjoyable
  - Plenaries
  - Invited talks
  - Posters
  - Mentoring/Science cafes/Meet the Editor
  - Pub quiz/Other socializing
2. The meeting element that I found LEAST enjoyable
  - Plenaries
  - Invited talks
  - Posters
  - Mentoring/Science cafes/Meet the Editor
  - Pub quiz/Other socializing
3. Did you encounter any technical problems? (tick any)
  - Looking at posters
  - Talking to posters
  - Engaging in social events
  - Asking questions
  - Watching Q&A
  - Walking talks
  - Log in
4. Please give details if applicable

[free text]

1. Did you use social media as part of the meeting (e.g., Twitter accounts) to follow content?
  - Yes
  - No
2. Did you use social media as part of the meeting (e.g., Twitter accounts) to advertise or disseminate content?
  - Yes
  - No
3. The current meeting was free. Should future ASAB meetings levy a charge?
  - Yes
  - No
4. Please explain your choice briefly

[free text]

1. If a future ASAB meeting were to charge, what would you consider to be an appropriate delegate fee for a day like today?
  - £0
  - £1–10
  - £11–25
  - £26–50
  - +£50
2. The virtual meeting was MORE attractive to me than a “real‐life” meeting because:
3. It was free
4. It permitted flexible participation during the day freeing me for other academic work
5. It permitted flexible participation during the day freeing me for other nonacademic work
6. It permitted flexible participation during the day freeing me for caring duties
7. Removed need to travel and reduced economic costs
8. Removed need to travel and reduced time costs
9. Removed need to travel and reduced carbon footprint
10. Easier to interact with other researchers and ask questions
11. More comfortable to interact with other researchers and ask questions
12. Access to a wider range of research work (more international)
  - Strongly agree
  - Agree
  - Somewhat agree
  - Either agree or disagree
  - Somewhat disagree
  - Disagree
  - Strongly disagree
13. The virtual meeting was LESS attractive to me than a “real‐life” meeting because:
14. Technical/computer issues
15. Time‐zone issues
16. Harder to interact with other researchers and ask questions
17. Harder to convey work in poster or talk format
18. Harder to interact socially with established colleagues
19. Harder to meet new colleagues
20. Lack of justification to visit another University/town/country as part of the conference trip
21. Frankly, just fed up of online meetings/zoom etc!
  - Strongly agree
  - Agree
  - Somewhat agree
  - Either agree or disagree
  - Somewhat disagree
  - Disagree
  - Strongly disagree
22. If you have any other comments about the day, good or bad, please add them below:

[free text]
